# Association between atherogenic index of plasma and hypertension: exploring the mediating role of body mass index in a Chinese population aged ≥ 45 years

**DOI:** 10.3389/fpubh.2025.1669033

**Published:** 2025-12-18

**Authors:** Liting Zhang, Lijuan Bai, Ruiyun Wang, Yun Liu, Man Liao, Jing Han, Chunyan Yang, Lihua Liu, Benling Qi

**Affiliations:** Department of Geriatrics, Union Hospital, Tongji Medical College, Huazhong University of Science and Technology, Wuhan, Hubei, China

**Keywords:** blood pressure, hypertension, atherogenic index of plasma, triglycerides, diabetes, mediation analysis

## Abstract

**Background:**

Atherosclerosis is recognized as a potential etiological factor for hypertension. However, evidence regarding the association between the Atherogenic Index of Plasma (AIP) and hypertension in Chinese middle-aged and older adults remains limited. This study aimed to examine the association between AIP and hypertension in this population.

**Methods:**

This retrospective single-center cross-sectional study consecutively enrolled 5,254 participants undergoing routine health examinations at the Health Management Center of Union Hospital Affiliated to Huazhong University of Science and Technology (Wuhan, China) between January 2017 and December 2019. Among them, 1,799 were diagnosed with hypertension and 579 with diabetes mellitus. The association between AIP and hypertension was analyzed using logistic regression and restricted cubic splines (RCS). Stratified analyses were performed by diabetes status. Furthermore, mediation analysis was conducted to evaluate the mediating effect of body mass index (BMI) on the AIP and hypertension association.

**Results:**

In this cross-sectional study of 5,254 participants, a positive association was observed between the atherogenic index of plasma (AIP) and hypertension. After adjusting for multifactorial confounders, each 1-unit increment in AIP was associated with a 14% higher odds of hypertension (aOR = 1.14, 95% CI: 1.02–1.27). Mediation analysis confirmed that body mass index (BMI) partially mediated this association, accounting for 55.62% of the total effect (*p* < 0.001).

**Conclusion:**

These findings suggest that elevated AIP is independently and positively associated with hypertension prevalence in adults aged ≥45 years, with body mass index (BMI) mediating 55.62% of this association (*p* < 0.001).

## 1 Introduction

As an important challenge in the global public health neighborhood, the incidence of hypertension continues to rise with the ageing of the population, making it an important causative factor for cardiovascular and cerebrovascular diseases, renal failure and other serious complications (2, 3). The development of hypertension is a multifactorial process, mainly due to a combination of factors such as genetics, environment and life style (4, 5). Globally, an estimated 1.39 billion adults had hypertension in 2010, of whom 349 million were in high-income countries and 1.04 billion in low-income countries (6). In China, hypertension contributes to a significant proportion of all cardiovascular disease-related deaths. Nearly half of Chinese adults aged 35–75 have hypertension (3). Therefore, proactive and effective hypertension control can prevent the onset and progression of cardiovascular and cerebrovascular diseases, playing a critical role in reducing their risks.

The Atherogenic Index of Plasma (AIP) was first proposed by Dobiásová as a new lipid index to assess the body’s susceptibility to atherosclerosis (7). AIP is a biomarker based on the calculation of the ratio of triglycerides (TG) to high-density lipoprotein cholesterol (HDL-C) in the blood, calculated using the formula log (TG/HDL-C). AIP has been shown to be strongly associated with atherosclerosis and cardiovascular disease risk (8, 9). Atherosclerosis is significantly affected by abnormalities in lipid metabolism, which may affect vascular function and blood pressure regulation through various pathways, leading to hypertension (1, 10). AIP demonstrates a unique bridging role in the complex network of metabolic disorders. This index breaks through the limitations of traditional lipid parameters, and can more sensitively reflect endothelial dysfunction and abnormal blood pressure regulation by quantifying the dynamic balance between atherogenic (TG) and anti-atherogenic (HDL-C) lipoproteins. Previous studies have shown that AIP is a reliable predictor of cardiovascular events, diabetes, metabolic syndrome, and obesity, suggesting its potential utility in assessing metabolic health (11–13).

While AIP is an established cardiovascular risk marker, its specific utility in the aging Chinese population—a demographic with distinct risk profiles—remains underexplored. More importantly, prior studies have largely treated body mass index (BMI) as a confounder, rather than examining its potential role as a mediator in the AIP-hypertension pathway. Therefore, this study aims not only to assess the association between AIP and hypertension in a large cohort of Chinese adults aged ≥45 years but also to quantitatively evaluate the mediating role of BMI, thereby providing novel mechanistic insights into this relationship.

## 2 Methods

### 2.1 Study design and data source

This is a retrospective cross-sectional study. This study utilised electronic health records from patients undergoing routine physical examinations at Union Hospital, Tongji Medical College, Huazhong University of Science and Technology, between January 2017 and December 2019. The extracted data analysed encompassed demographic characteristics, clinical laboratory test results, and physician-diagnosed medical conditions. All methods were performed following relevant guidelines and regulations, and all key aspects of the study are reported. Patients’ data were maintained confidentially. The research adheres to the Declaration of Helsinki and received ethical approval from the Ethics Committee of Union Hospital, Tongji Medical College, Huazhong University of Science and Technology on September 4th, 2023 ([2023]ID:0611). The need for informed consent was waived by the ethics committee.

### 2.2 Study population and selection criteria

**Figure 1:**
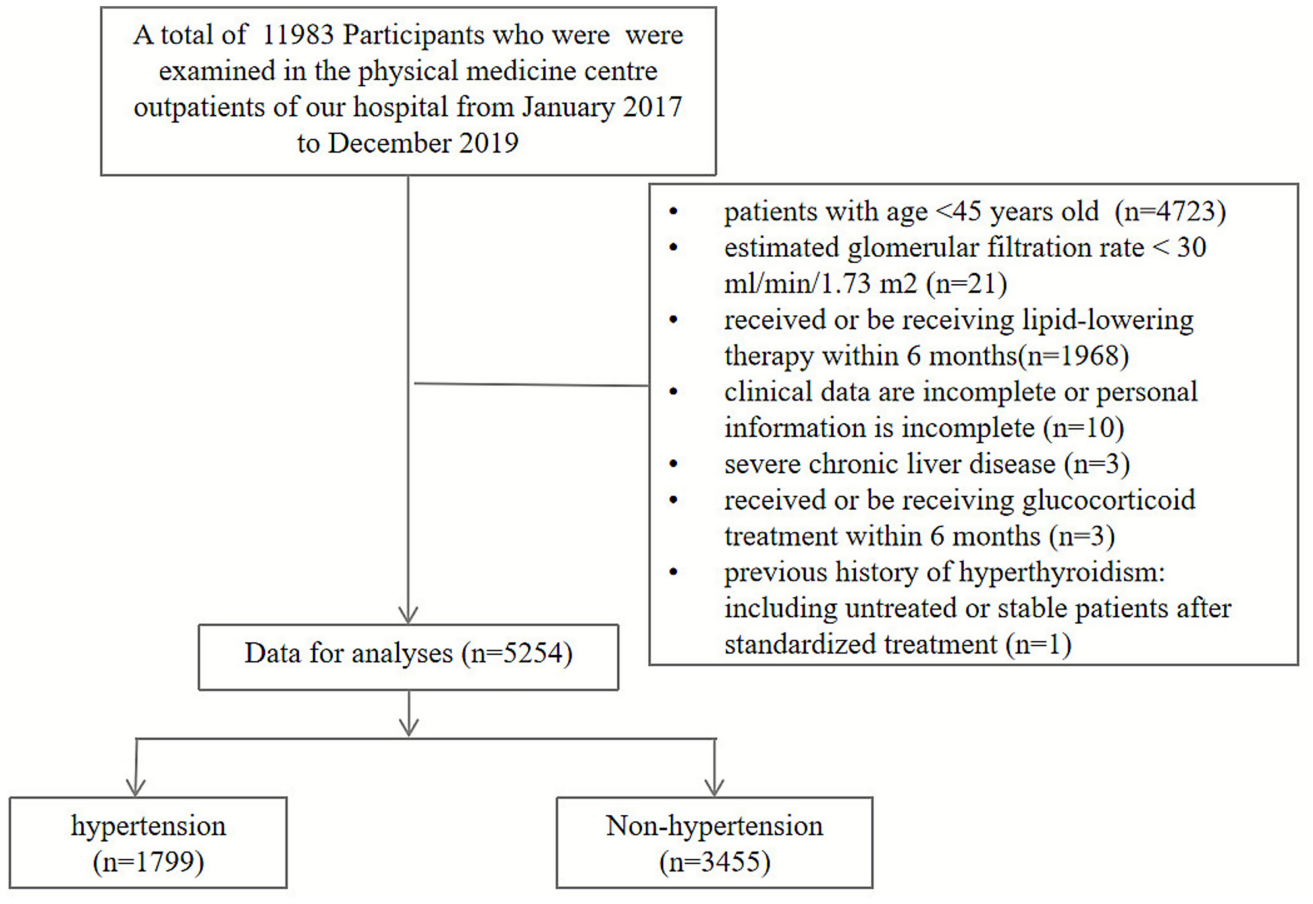
Participant screening and inclusion flowchart.

This study initially enrolled 11,983 individuals who underwent health examinations at our medical center between January 2017 and December 2019. Information identifying individual participants was not available to the authors during or after data collection. After applying the exclusion criteria: (1) age <45 years; (2) received lipid-lowering therapy within the past 6 months, including but not limited to statins, fibrates, ezetimibe, or PCSK9 inhibitors; (3) estimated glomerular filtration rate (eGFR) < 30 mL/min/1.73m^2^; (4) severe chronic liver disease; (5) history of hyperthyroidism (including untreated cases or those stabilized with standardized treatment); (6) current or recent (within 6 months) glucocorticoid therapy; and (7) incomplete clinical or demographic data. After screening, the final study population comprised 5,254 participants. Among them, 1,799 were categorized into the hypertension group and 3,455 into the non-hypertension group based on their documented diagnosis at the time of examination. The participant selection flow chart is detailed in Figure 1.

### 2.3 Ascertainment of AIP

The Atherogenic Index of Plasma (AIP), a composite metric reflecting lipid metabolism status, quantifies atherosclerosis risk through logarithmic transformation of the triglyceride to high-density lipoprotein cholesterol ratio (AIP = log [TG/HDL-C]). In subsequent analyses, AIP was first examined as a continuous variable. To enhance analytical rigor, participants were stratified into tertiles based on AI *p* values: T1 (AIP < −0.26), T2 (−0.26 ≤ AIP < 0.34), and T3 (AIP ≥ 0.34).

### 2.4 Ascertainment of hypertension and type 2 diabetes mellitus

The primary outcome of this study was the prevalence of hypertension. Hypertension was defined as meeting at least one of the following criteria: (1) mean systolic blood pressure ≥140 mmHg and/or mean diastolic blood pressure ≥90 mmHg; (2) self-reported hypertension history; or (3) current antihypertensive medication use.

Type 2 diabetes mellitus (T2DM) was defined according to standard criteria: (1) fasting plasma glucose ≥7 mmol/L; (2) glycated hemoglobin >6.5%; (3) self-reported history of diabetes; or (4) current use of glucose-lowering agents.

### 2.5 Ascertainment of covariates

Age, sex, height, and weight were retrieved from the hospital electronic system, with body mass index (BMI) calculated as weight in kilograms divided by height in meters squared (kg/m^2^). After an overnight fast, venous blood samples were drawn by certified nurses and analyzed in the laboratory for biochemical parameters including: aspartate aminotransferase (AST), triglycerides (TG), total cholesterol (TC), high-density lipoprotein cholesterol (HDL-C), low-density lipoprotein cholesterol (LDL-C), uric acid (UA), blood urea nitrogen (BUN), and creatinine. Estimated glomerular filtration rate (eGFR) was derived using the modified Modification of Diet in Renal Disease (MDRD) equation. Comorbidities, smoking history, and alcohol consumption were obtained from medical records or self-reports. Smoking history was defined as a binary variable (yes/no), where “yes” encompassed both current and former smokers. Drinking history was defined as a binary variable (yes/no), based on whether the participant had a history of alcohol consumption.

### 2.6 Statistical analysis

Normality of continuous variables was assessed using the Shapiro–Wilk test. Normally distributed continuous data were expressed as mean ± standard deviation (SD) and comparisons between groups were made using the independent samples t-test or one-way ANOVA. Non-normally distributed continuous data were expressed as median and interquartile range (IQR), and comparisons were made using the Kruskal-Wallis test. Categorical data were expressed as numbers and percentages (%) and compared between groups using the Chi-square test or Fisher’s exact test, as appropriate.

The association between the atherogenic index of plasma (AIP) and hypertension was assessed using a series of multivariable logistic regression models. The results are presented as odds ratios (ORs) with corresponding 95% confidence intervals (CIs). To transparently account for potential confounding factors, we employed the following sequential adjustment strategy: Crude model: Adjusted for no covariates. Model 1: Adjusted for basic demographic and anthropometric factors: age, sex, and body mass index. Model 2: Further adjusted for a comprehensive set of potential confounders, which included lifestyle factors such as smoking history and drinking history; clinical comorbidities including diabetes mellitus, hyperuricemia, osteoporosis, and carotid artery stenosis; renal function parameters such as estimated glomerular filtration rate, blood urea nitrogen, and creatinine; as well as other cardiometabolic parameters including aspartate aminotransferase, low-density lipoprotein cholesterol, albumin/globulin ratio, and glycated hemoglobin. This model represents our most rigorous adjustment. A two-sided *p*-value < 0.05 was considered statistically significant. All statistical analyses were performed using R software (version 4.3.2).

## 3 Result

### 3.1 Clinical baseline data

The study included 5,254 participants stratified by AIP tertiles: T1 (AIP < −0.26, *n* = 1,734), T2 (−0.26 ≤ AIP < 0.34, *n* = 1,784), and T3 (AIP ≥ 0.34, *n* = 1,736). Baseline characteristics are summarized in Table 1. Blood urea nitrogen (BUN) and carotid atherosclerosis (CAS) prevalence showed no significant differences across tertiles (*p* > 0.05). In contrast, statistically significant intergroup differences (*p* < 0.05) were observed in: age, sex, body mass index (BMI), creatinine, estimated glomerular filtration rate (eGFR), uric acid (UA), high-density lipoprotein cholesterol (HDL-C), low-density lipoprotein cholesterol (LDL-C), triglycerides (TG), aspartate aminotransferase (AST), hypertension prevalence, diabetes prevalence, smoking history, and alcohol consumption history.

**Table 1:** Baseline characteristics stratified by atherogenic index of plasma (AIP) tertiles.

	AIP				
Characteristics	Total	T1 (<−0.26)	T2 (−0.26 ~ 0.34)	T3 (>0.34)	*p*
	(*n* = 5,254)	(*n* = 1734)	(*n* = 1784)	(*n* = 1736)	
Age, year	59.59 ± 10.85	60.44 ± 11.59	60.22 ± 10.72	58.11 ± 10.03	<0.001
Albumin, g/L	46.46 ± 2.51	46.22 ± 2.55	46.34 ± 2.48	46.83 ± 2.48	<0.001
Globulin, g/L	27.63 ± 3.57	27.41 ± 3.63	27.75 ± 3.57	27.73 ± 3.49	0.007
AGR	1.71 ± 0.24	1.72 ± 0.25	1.70 ± 0.24	1.72 ± 0.24	0.045
AST, U/L	24.80 ± 16.55	20.66 ± 11.29	24.48 ± 17.39	29.26 ± 18.82	<0.001
TG, mmol/L	1.71 ± 1.20	0.88 ± 0.21	1.41 ± 0.29	2.84 ± 1.46	<0.001
HDL_C, mmol/L	1.39 ± 0.34	1.68 ± 0.34	1.35 ± 0.22	1.14 ± 0.20	<0.001
LDL_C, mmol/L	2.87 ± 0.76	2.69 ± 0.69	2.99 ± 0.75	2.92 ± 0.79	<0.001
BUN, mmol/L	5.31 ± 1.35	5.32 ± 1.33	5.36 ± 1.36	5.26 ± 1.36	0.120
Cr, umol.L	75.78 ± 16.16	72.56 ± 15.62	76.27 ± 16.14	78.48 ± 16.17	<0.001
eGFR, mL/min/1.73 m2	98.12 ± 20.63	100.56 ± 21.08	97.55 ± 20.57	96.26 ± 20.00	<0.001
Uric acid, umol/L	367.88 ± 88.90	330.43 ± 80.75	369.55 ± 82.65	403.57 ± 87.83	<0.001
HbA1C	5.76 ± 0.84	5.63 ± 0.71	5.74 ± 0.79	5.90 ± 0.98	<0.001
BMI, kg/m2	24.44 ± 3.03	22.96 ± 2.78	24.65 ± 2.89	25.71 ± 2.76	<0.001
Sex, *n*(%)					<0.001
Male	3,904 (74.31)	1,089 (62.80)	1,358 (76.12)	1,457 (83.93)	
Female	1,350 (25.69)	645 (37.20)	426 (23.88)	279 (16.07)	
Osteoporosis, *n*(%)					<0.001
No	3,880 (73.85)	1,220 (70.36)	1,350 (75.67)	1,310 (75.46)	
Yes	966 (18.39)	381 (21.97)	304 (17.04)	281 (16.19)	
Hypertension, *n*(%)					<0.001
No	3,455 (65.76)	1,237 (71.34)	1,167 (65.41)	1,051 (60.54)	
Yes	1799 (34.24)	497 (28.66)	617 (34.59)	685 (39.46)	
CAS, *n*(%)					0.634
No	3,419 (65.07)	1,123 (64.76)	1,151 (64.52)	1,145 (65.96)	
Yes	1835 (34.93)	611 (35.24)	633 (35.48)	591 (34.04)	
Diabetes, *n*(%)					<0.001
No	4,675 (88.98)	1,613 (93.02)	1,602 (89.80)	1,460 (84.10)	
Yes	579 (11.02)	121 (6.98)	182 (10.20)	276 (15.90)	
Hyperuricemia, *n*(%)					<0.001
No	2,474 (47.09)	1,141 (65.80)	813 (45.57)	520 (29.95)	
Yes	2,780 (52.91)	593 (34.20)	971 (54.43)	1,216 (70.05)	
Smoking, *n*(%)					<0.001
No	3,208 (61.06)	1,182 (68.17)	1,078 (60.43)	948 (54.61)	
Yes	2046 (38.94)	552 (31.83)	706 (39.57)	788 (45.39)	
Drinking, *n*(%)					0.032
No	3,816 (72.63)	1,297 (74.80)	1,265 (70.91)	1,254 (72.24)	
Yes	1,438 (27.37)	437 (25.20)	519 (29.09)	482 (27.76)	

aspartate aminotransferase, AST; albumin/globulin ratio, AGR; body mass index, BMI; carotid artery stenosis, CAS; Hemoglobin A1c, HbA1C;triglycerides, TG; high density lipoprotein cholesterol, HDL_C; low density lipoprotein cholesterol, LDL_C; blood urea nitrogen, BUN; creatinine, Cr; estimated glomerular filtration rate, eGFR.

### 3.2 Binary logistic regression analysis of hypertension

**Figure 2:**
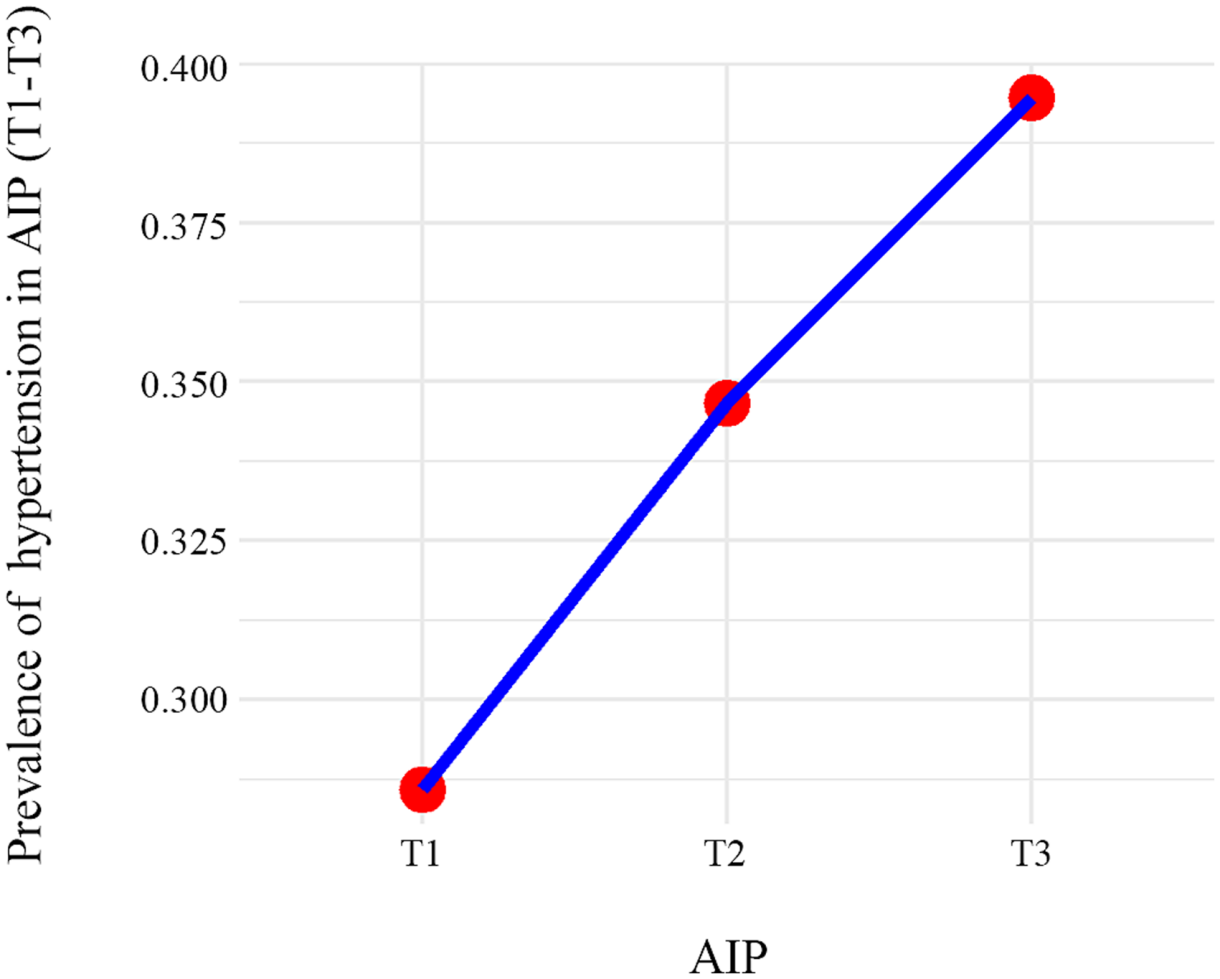
Prevalence of hypertension in AIP (T1-T3).

The association of AIP with hypertension was evaluated using both univariable and multivariable logistic regression, treating AIP as a continuous variable and in tertiles (T1-T3). In univariable analysis, both parameterizations of AIP showed significant positive associations with hypertension (all *p* < 0.001). These associations remained significant after sequential adjustment for confounders (Table 2). In the fully adjusted model (Model 2), each unit increase in continuous AIP was associated with a 14% higher odds of hypertension (aOR = 1.14, 95% CI: 1.02–1.27). A significant dose–response relationship was observed across tertiles (P for trend = 0.031), with participants in the highest tertile (T3) having a 22% increased odds of hypertension compared to the lowest tertile (T1) (aOR = 1.22, 95% CI: 1.02–1.47). Notably, the T3 group exhibited significantly higher odds of hypertension compared to the T1 reference group, as visually depicted in Figure 2. The trend was visualized by calculating the mean prevalence of hypertension within each AIP tertile and connecting these points with a line.

**Table 2:** Logistic regression models for predicting the incidence of hypertension according to AIP.

Variable	Crude model	Model 1	Model 2
	OR (95%CI)	OR (95%CI)	OR (95%CI)
AIP	1.34 (1.24 ~ 1.46)	1.20 (1.09 ~ 1.32)	1.14 (1.02 ~ 1.27)
*p*-value	0.000	0.001	0.021
T1	Ref	Ref	Ref
T2	1.32 (1.14 ~ 1.52)	1.08 (0.93 ~ 1.27)	1.09 (0.92 ~ 1.29)
T3	1.62 (1.41 ~ 1.87)	1.32 (1.13 ~ 1.55)	1.22 (1.02 ~ 1.47)
*P* for trend	0.000	0.001	0.031

Crude model adjusted for none. Model 1 adjusted for age, sex, and body mass index. Model 2 further adjusted for aspartate aminotransferase (AST), estimated glomerular filtration rate (eGFR), low density lipoprotein cholesterol (LDL_C), blood urea nitrogen (BUN), creatinine, albumin/globulin ratio (AGR), glycated hemoglobin (HbA1c), osteoporosis, diabetes, hyperuricemia, carotid artery stenosis (CAS), drinking history, and smoking history in addition to covariates in Model 1.

### 3.3 Restricted cubic splines

**Figure 3:**
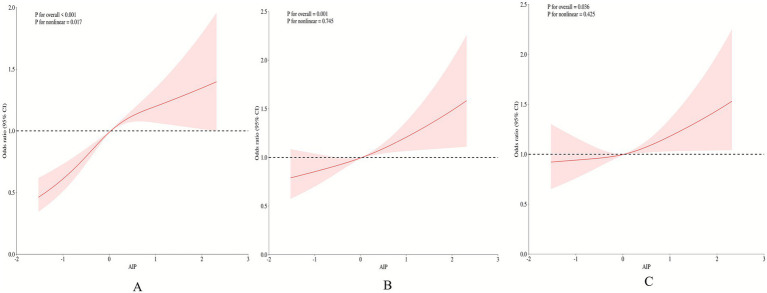
Restricted cubic spline analysis of AIP and hypertension association. **(A)** Crude model adjusted for none. **(B)** Adjusted for age, sex, and body mass index. **(C)** adjusted for age, sex, body mass index, aspartate aminotransferase (AST), blood urea nitrogen (BUN), estimated glomerular filtration rate (eGFR), low density lipoprotein cholesterol (LDL_C), creatinine, albumin/globulin ratio (AGR), glycated hemoglobin (HbA1c), drinking history, smoking history, carotid artery stenosis (CAS), osteoporosis, diabetes, and hyperuricemia.

To evaluate potential nonlinear relationships between AIP and hypertension prevalence, restricted cubic splines (RCS) were fitted (Figure 3). Notably, a J-shaped curve was observed in the unadjusted model (P for nonlinear = 0.017). After adjusting for relevant covariates, the nonlinear association was attenuated (P for nonlinear = 0.425), while the overall association remained statistically significant (P for overall = 0.036), indicating a predominantly linear dose–response relationship (Figure 3C).

### 3.4 Logistic regression analysis of AIP and hypertension in patients with diabetes mellitus

**Figure 4:**
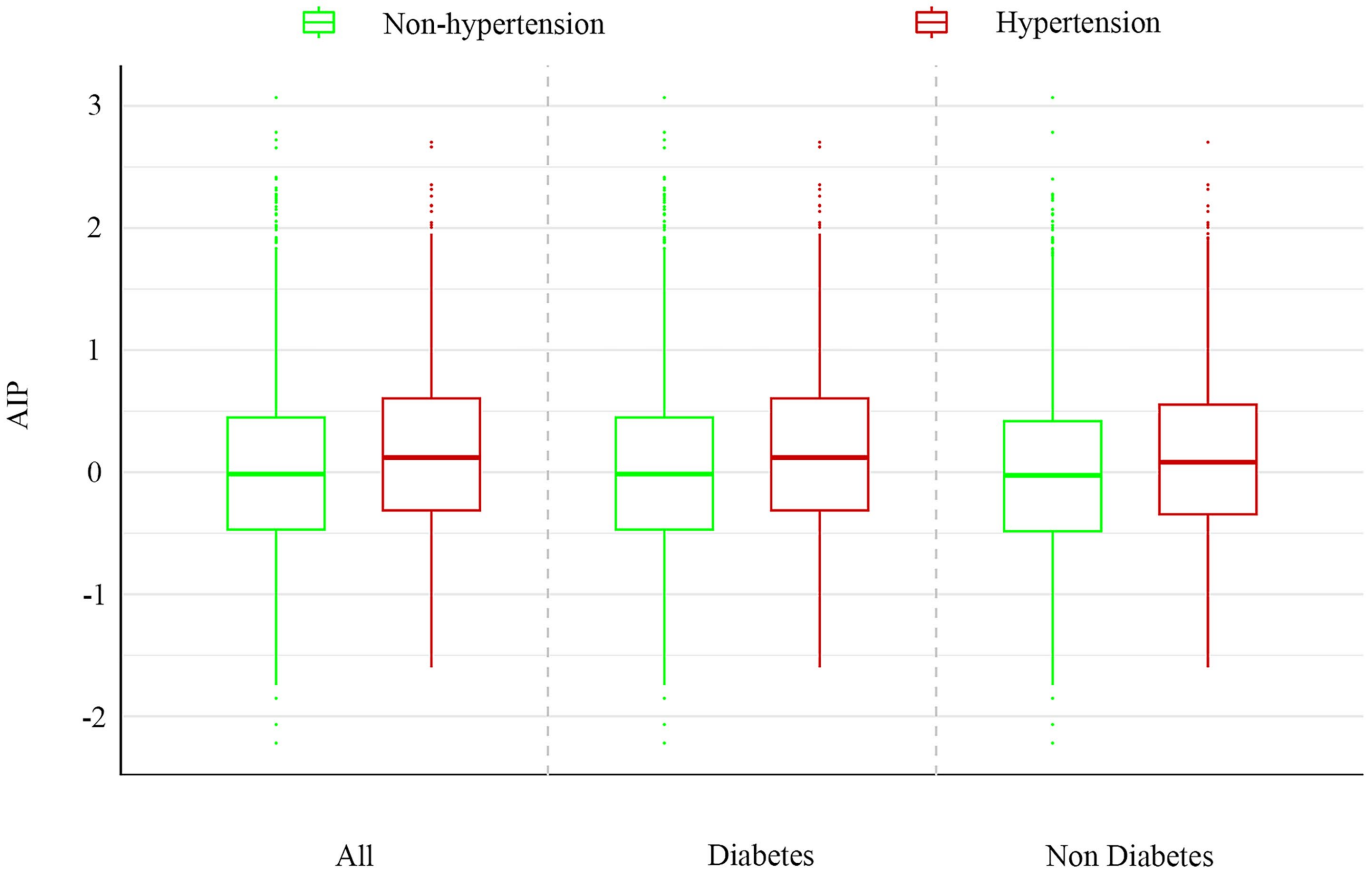
Distribution differences of AIP between diabetic and non-diabetic populations.

The study included 579 diabetic patients (11.02%) and 4,675 non-diabetic individuals (88.98%). Stratified analyses by diabetes status were performed (Table 3). After full adjustment for covariates (Model 2), the highest tertile of AIP (T3) was significantly associated with increased odds of hypertension compared to the lowest tertile (T1) in both diabetic and non-diabetic populations. Notably, the association was stronger in individuals with diabetes (aOR = 1.72, 95% CI: 1.08–2.75) than in those without (aOR = 1.19, 95% CI: 1.01–1.42). A significant positive trend across AIP tertiles was observed in both groups (P for trend < 0.05). The distribution of AIP values across the cohorts is shown in Figure 4.

**Table 3:** Logistic regression analysis of AIP to predict the risk of hypertension in diabetes and non-diabetes.

Characteristics	Crude model		Model 1		Model 2	
	OR (95%CI)	*P* value	OR (95%CI)	*P* value	OR (95%CI)	*P* value
Diabetes						
AIP	1.21 (0.96 ~ 1.53)	0.101	1.27 (0.99 ~ 1.64)	0.065	1.40 (1.07 ~ 1.84)	0.014
T1	Ref		Ref		Ref	
T2	1.46 (0.98 ~ 2.18)	0.064	1.66 (1.09 ~ 2.51)	0.017	1.42 (0.92 ~ 2.19)	0.116
T3	1.53 (1.02 ~ 2.29)	0.041	1.96 (1.28 ~ 3.01)	0.002	1.72 (1.08 ~ 2.75)	0.028
P for trend	0.035		0.049		0.023	
Non-diabetes						
AIP	1.31 (1.20 ~ 1.43)	0.000	1.16 (1.05 ~ 1.29)	0.005	1.12 (1.01 ~ 1.25)	0.041
T1	ref		ref		ref	
T2	1.34 (1.15 ~ 1.57)	0.000	1.09 (0.92 ~ 1.29)	0.306	1.08 (0.92 ~ 1.28)	0.347
T3	1.52 (1.31 ~ 1.77)	0.000	1.22 (1.03 ~ 1.45)	0.024	1.19 (1.01 ~ 1.42)	0.049
P for trend	0.000		0.023		0.048	

Crude model adjusted for none. Model 1 adjusted for age, sex, and body mass index. Model 2 further adjusted for aspartate aminotransferase (AST), estimated glomerular filtration rate (eGFR), low density lipoprotein cholesterol (LDL_C), blood urea nitrogen (BUN), creatinine, albumin/globulin ratio (AGR), osteoporosis, hyperuricemia, carotid artery stenosis (CAS), drinking history, and smoking history in addition to covariates in Model 1.

### 3.5 Mediation analysis of BMI on associations of AIP with hypertension

**Figure 5:**
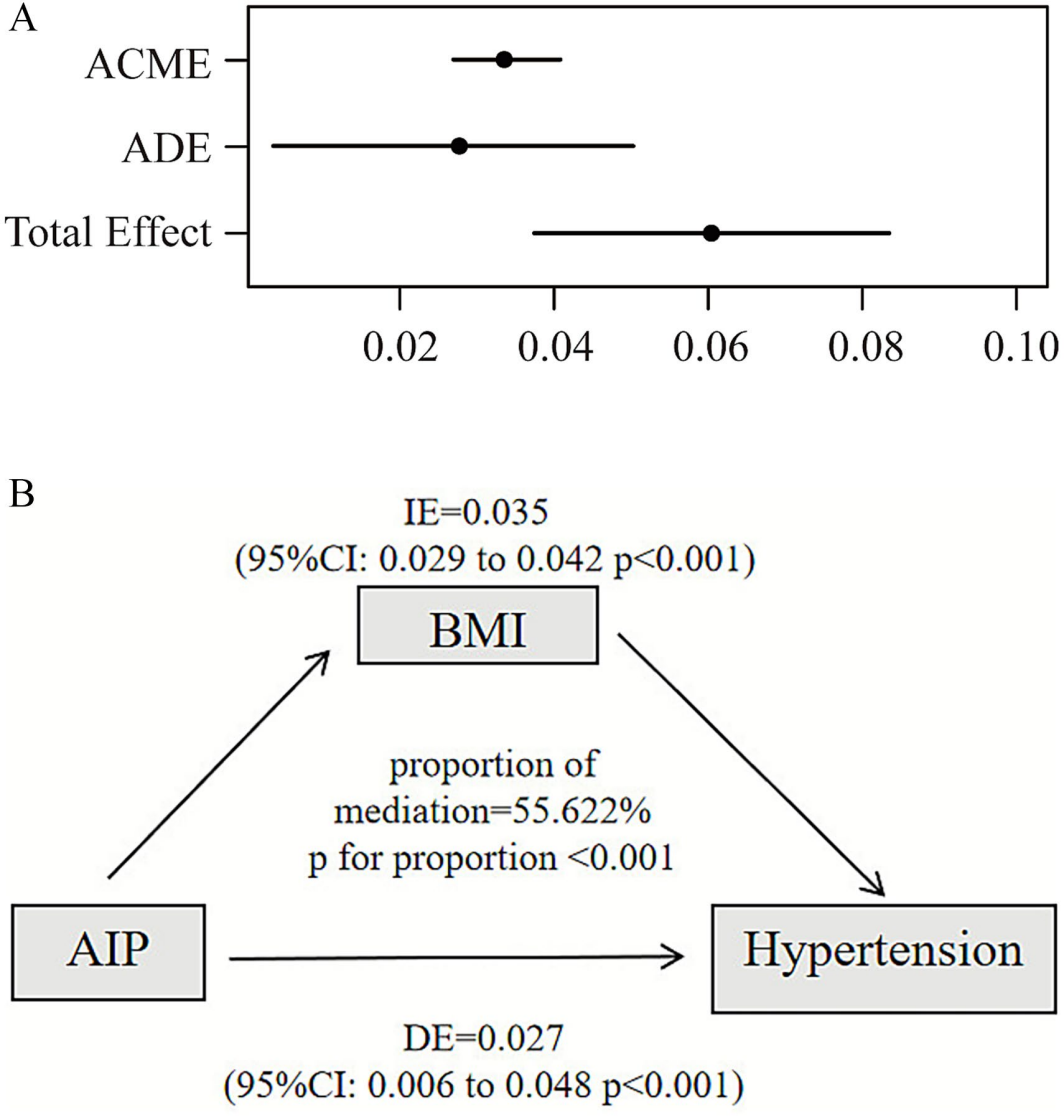
Mediating role of BMI in the association between AIP and hypertension. **(A)** Mediation regression model. **(B)** Directed acyclic graph (DAG) illustrating mediation pathways. ACME: Average Causal Mediation Effect (indirect effect); ADE: Average Direct Effect (direct effect); Total Effect: Combined direct and indirect effect. Proportion Mediated: Percentage of the total correlation between AIP and hypertension explained by the mediating pathway through BMI. Adjust: age, sex, aspartate aminotransferase (AST), estimated glomerular filtration rate (eGFR), low density lipoprotein cholesterol (LDL_C), blood urea nitrogen (BUN), creatinine, albumin/globulin ratio (AGR), osteoporosis, hyperuricemia, carotid artery stenosis (CAS), drinking history, and smoking history.

As in Figure 5, mediation analyses showed direct and indirect effects between AIP and hypertension after adjusting for covariates [estimate (95% CI): DE = 0.027 (0.006, 0.048), IE = 0.035 (0.029, 0.042)]. The indirect effect of AIP on HTN via BMI was significant (*p* < 0.001), accounting for 55.622% of the total effect.

## 4 Discussion

This study used medical record data from patients attending the medical check-up clinic at the Union Hospital in Wuhan, China, to investigate the association between AIP and hypertension in middle-aged and older adults, with stratified subgroup analyses in both diabetic and non-diabetic populations. In this study of a Chinese middle-aged and older population, we noted that the prevalence of hypertension was significantly higher among participants in the high AIP group in the baseline data (AIP ≥ 0.34, *p* < 0.001). After adjusting for covariates that may influence hypertension, including age, sex, smoking and drinking status, BMI, LDL-C, BUN, eGFR, and so on, we found a still-significant association between high AIP and the prevalence of hypertension. Patients with AIP >0.34 (T3) had a significantly increased risk of hypertension compared with those with AIP < −0.26 (T1). In addition, this study evaluated the significance value of AIP index in predicting hypertension, especially in diabetic patients, providing a new risk indicator for identifying patients with asymptomatic hypertension combined with diabetes mellitus. The results showed that the risk of hypertension in the high AIP group was still higher than that in the low AIP group in both groups, and the statistical analysis was significant. Finally, we performed further mediation analyses and showed that the underlying biological mechanisms of AIP-associated hypertension in cross-sectional studies were partially attributable to the increase in BMI.

AIP is considered a validated marker for early diagnosis of atherosclerosis and CVD, and previous studies have focused on the association between AIP and CVD, acute myocardial infarction or cardiovascular metabolic diseases (14, 15). A cross-sectional study utilising data from the China Health and Retirement Longitudinal Study identified a significant association between AIP and physical functional impairment in older adults (16). A retrospective cohort study using the National Health and Nutrition Examination Survey (NHANES) database found that AIP was associated with all-cause mortality and cardiovascular disease (CVD) in hypertensive patients (17). Another study using data from NHANES 2005–2018 examined a J-shaped relationship between baseline AIP levels and cardiovascular disease mortality and all-cause mortality in the US adult population, particularly among individuals aged 40–60 years (18). A study of Japanese adults aged ≥18 years demonstrated that a higher AIP was significantly associated with an increased risk of prehypertension or hypertension in normoglycemic participants from Gifu, Japan. This association was more pronounced in women, particularly those aged 40–60 years (19). In another study by Chinese scholars, adult individuals with higher AIP indices were found to be associated with new-onset hypertension, with a stronger correlation observed in subjects with a normal BMI (20). The above findings are in line with the conclusions of our study. However, few studies have examined the relationship between AIP and hypertension in middle-aged and older adults. We conclude that AIP is a novel serum marker closely related to hypertension, which could be beneficial in the prevention and treatment of cardiovascular disease and its complications if lipid control is made a condition for preventing the development of hypertension.

The biological mechanisms linking elevated AIP to increased hypertension prevalence remain unclear. Potential explanations include: First, the endothelium serves as the primary regulator of vascular tone and a key contributor to salt sensitivity through nitric oxide (NO) (21). NO, produced by endothelial cells, represents the most critical vasoactive substance for blood pressure regulation (22, 23). Both high TG and low HDL-C reduce NO bioavailability, which directly impairs vascular endothelium-dependent diastolic function and leads to increased vascular tone (24). Second, elevated AIP is often accompanied by a chronic low-grade inflammatory state (25). Inflammatory factors such as TNF-a and IL-6 can also impair vascular endothelial function, promote vasoconstriction and remodeling, and elevate blood pressure (25). Third, AIP affects the occurrence of hypertension and insulin resistance is closely associated with insulin resistance, insulin resistance lipids manifested in high triglycerides and low HDL cholesterol, elevated AIP is the existence of insulin resistance is sensitive and important markers, high AIP largely suggests that there is a potential state of insulin resistance (26, 27). In insulin resistance, pancreatic *β*-cells compensatorily secrete more insulin in an attempt to maintain normal blood glucose, resulting in a sustained elevation of circulating insulin levels, and this chronic hyperinsulinaemia activates the sympathetic nervous system, promotes renal water and sodium reabsorption, activates the renin-angiotensin-aldosterone system (RASS), and enhances vascular reactivity to vasoconstrictive substances, which together result in an increase in blood pressure (28, 29). Visceral adipose tissue is a major site for the production of free fatty acids, which enter the liver to promote very low-density lipoprotein (VLDL) synthesis and thus TG elevation (30). Moreover, high AIP is often closely associated with the accumulation of visceral fat, and high AIP is both a consequence of visceral obesity and may further exacerbate obesity and its complications by promoting ectopic fat deposition and metabolic disturbances (31).

Diabetes and hypertension share common pathophysiological foundations, notably insulin resistance and hyperinsulinemia, which can lead to sympathetic nervous system activation, renal sodium retention, and endothelial dysfunction, thereby increasing hypertension risk. Furthermore, diabetes is often accompanied by more severe atherogenic dyslipidemia, which may synergistically contribute to hypertension development (32). We hypothesized that the AIP-hypertension association would be stronger in individuals with diabetes. Our results, which showed a more pronounced positive association in the diabetic subgroup, confirm this hypothesis, suggesting that the impact of dyslipidemia on blood pressure is amplified in the context of impaired glucose metabolism.

Our mediation analysis indicates that BMI partially mediates the relationship between AIP and hypertension, providing crucial mechanistic insights into the intrinsic link between lipid metabolism and blood pressure. Based on the aforementioned biological mechanisms, we propose the following pathway hypothesis: the atherogenic lipid profile represented by AIP first promotes visceral fat accumulation and central obesity characterised by BMI, with obesity subsequently driving hypertension through the following core mechanisms. First, visceral adipose tissue releases free fatty acids and pro-inflammatory cytokines while reducing adiponectin levels, triggering systemic low-grade inflammation and oxidative stress. This leads to endothelial dysfunction and diminished nitric oxide bioavailability (33, 34). Second, visceral obesity exacerbates insulin resistance, activating the RAAS system and sympathetic nervous system, thereby promoting renal sodium reabsorption and vascular remodeling (35). Finally, obesity itself increases cardiac output and blood volume, amplifying mechanical stress on vascular walls (36). Consequently, BMI is not merely a confounding factor but a pivotal intermediate phenotype within the causal pathway linking AIP to hypertension. Our study confirms BMI’s partial mediating role in this pathway, underscoring the critical importance of weight management in regulating the relationship between lipid metabolism and hypertension. This study possesses several strengths, including a substantial sample size derived from reliable real-world data, which enhances the reliability of our findings. Furthermore, the stability of the core association was corroborated through subgroup analyses stratified by diabetes status. The primary contribution of this work extends beyond replicating this association in a middle-aged and older Chinese cohort—a previously understudied demographic. Most importantly, by employing causal mediation analysis, we moved beyond the conventional approach of treating BMI as a confounder and instead conceptualized and demonstrated it as a significant mediator. This pivotal shift in perspective, from confounding to mediation, provides a more integrated understanding of the interplay between dyslipidemia, obesity, and hypertension. However, there are still some limitations of this study, firstly, this is a cross-sectional study, and it is difficult to determine the causal relationship between AIP and prevalence of hypertension in a cross-sectional study. Secondly, although we have adjusted for most of the relevant confounders, there may still be relevant unknown confounders that were not included, such as detailed dietary patterns, which we were unable to account for. Most importantly, our study sample was drawn from a specific population in central China (Wuhan). Although the sample size was substantial and well-characterized, the external validity of our results when generalized to other regions of China or to populations in other countries may be limited. China is a vast country with disparities in socioeconomic status, dietary habits, and healthcare levels across different regions. Furthermore, other countries have entirely different population genetic backgrounds and disease spectra. Therefore, our conclusions should be validated in prospective cohort studies involving diverse geographical and ethnic populations before being applied to broader contexts.

## 5 Conclusion

The results showed that AIP was significantly and positively associated with the prevalence of hypertension in the middle-aged and older people, and this relationship is partially mediated by BMI. Our findings highlight the importance of weight management as a potential target for mitigating the risk of hypertension associated with atherogenic dyslipidemia.

## Data Availability

The raw data supporting the conclusions of this article will be made available by the authors, without undue reservation.
