# Supplementary material for: Association between atherogenic index of plasma and hypertension: exploring the mediating role of body mass index in a Chinese population aged ≥ 45 years
Source: Front Public Health. 2025 Dec 18;13:1669033. doi: 10.3389/fpubh.2025.1669033 (PMC12756139; doi:10.3389/fpubh.2025.1669033)
Supplement: Supplementary file 1 [file Table_1.docx]

Table1S Baseline characteristics between hypertension and non- hypertension

| Variables | Total  (n = 5254) | Non-hypertension  (n = 3455) | Hypertension  (n = 1799) | *P* |
| --- | --- | --- | --- | --- |
|  |  |  |  |  |
| Age,,year | 59.59 ± 10.85 | 57.68 ± 10.03 | 63.27 ± 11.40 | <.001 |
| **Albumin**,g/L | 46.46 ± 2.51 | 46.41 ± 2.50 | 46.55 ± 2.55 | 0.060 |
| Globulin,g/L | 27.63 ± 3.57 | 27.43 ± 3.54 | 28.03 ± 3.59 | <.001 |
| AGR | 1.71 ± 0.24 | 1.72 ± 0.24 | 1.69 ± 0.24 | <.001 |
| AST,U/L | 24.80 ± 16.55 | 24.42 ± 16.69 | 25.54 ± 16.25 | 0.020 |
| TG,mmol/L | 1.71 ± 1.20 | 1.65 ± 1.17 | 1.83 ± 1.24 | <.001 |
| HDL_C,mmol/L | 1.39 ± 0.34 | 1.41 ± 0.35 | 1.35 ± 0.32 | <.001 |
| LDL_C,mmol/L | 2.87 ± 0.76 | 2.86 ± 0.76 | 2.88 ± 0.76 | 0.432 |
| BUN,mmol/L | 5.31 ± 1.35 | 5.25 ± 1.30 | 5.44 ± 1.43 | <.001 |
| Cr,umol.L | 75.78 ± 16.16 | 74.64 ± 15.47 | 77.96 ± 17.20 | <.001 |
| eGFR,mL/min/1.73 m2 | 98.12 ± 20.63 | 99.50 ± 20.18 | 95.47 ± 21.21 | <.001 |
| Uric acid,umol/L | 367.88 ± 88.90 | 360.47 ± 88.29 | 382.11 ± 88.35 | <.001 |
| HbA1C | 5.76 ± 0.84 | 5.69 ± 0.81 | 5.88 ± 0.90 | <.001 |
| BMI, kg/m2 | 24.44 ± 3.03 | 24.02 ± 2.93 | 25.27 ± 3.04 | <.001 |
| Osteoporosis, n(%) |  |  |  | 0.050 |
| NO | 3880 (73.85) | 2568 (74.33) | 1312 (72.93) |  |
| Yes | 966 (18.39) | 606 (17.54) | 360 (20.01) |  |
| Sex, n(%) |  |  |  | <.001 |
| male | 3904 (74.31) | 2458 (71.14) | 1446 (80.38) |  |
| female | 1350 (25.69) | 997 (28.86) | 353 (19.62) |  |
| CAS, n(%) |  |  |  | <.001 |
| NO | 3419 (65.07) | 2435 (70.48) | 984 (54.70) |  |
| Yes | 1835 (34.93) | 1020 (29.52) | 815 (45.30) |  |
| Diabetes, n(%) |  |  |  | <.001 |
| NO | 4675 (88.98) | 3154 (91.29) | 1521 (84.55) |  |
| Yes | 579 (11.02) | 301 (8.71) | 278 (15.45) |  |
| Hyperuricemia,  , n(%) |  |  |  | <.001 |
| NO | 2474 (47.09) | 1740 (50.36) | 734 (40.80) |  |
| Yes | 2780 (52.91) | 1715 (49.64) | 1065 (59.20) |  |
| Smoking, n(%) |  |  |  | <.001 |
| NO | 3208 (61.06) | 2229 (64.52) | 979 (54.42) |  |
| Yes | 2046 (38.94) | 1226 (35.48) | 820 (45.58) |  |
| Drinking, n(%) |  |  |  | 0.012 |
| NO | 3816 (72.63) | 2548 (73.75) | 1268 (70.48) |  |
| Yes | 1438 (27.37) | 907 (26.25) | 531 (29.52) |  |

Abbreviations: aspartate aminotransferase,AST;albumin/globulin ratio,AGR; body mass index,BMI;carotid artery stenosis,CAS;Hemoglobin A1c,HbA1C;t**riglycerides,**TG;**high density lipoprotein cholesterol,**HDL_C;low density lipoprotein cholesterol,LDL_C;blood urea nitrogen,BUN;creatinine,Cr;estimated glomerular filtration rate,eGFR.
